# Improving physical activity in hospitalized patients: The preliminary effectiveness of a goal-directed movement intervention

**DOI:** 10.1177/02692155231189607

**Published:** 2023-07-24

**Authors:** JWM van Grootel, P Bor, JA Netjes, C Veenhof, K Valkenet

**Affiliations:** 1 Department of Rehabilitation, Physiotherapy Science & Sport, UMC Utrecht Brain Center, 8124 University Medical Center Utrecht, Utrecht, Utrecht, The Netherlands; 2 Research Center of Healthy and Sustainable Living, Research group Innovation of Movement Care, 8119 HU University of Applied Sciences Utrecht, Utrecht, Utrecht, The Netherlands

**Keywords:** Intervention, hospitalization, accelerometer, physical activity, goal setting

## Abstract

**Objective:**

To evaluate the preliminary effectiveness of a goal-directed movement intervention using a movement sensor on physical activity of hospitalized patients.

**Design:**

Prospective, pre-post study.

**Setting:**

A university medical center.

**Participants:**

Patients admitted to the pulmonology and nephrology/gastro-enterology wards.

**Intervention:**

The movement intervention consisted of (1) self-monitoring of patients’ physical activity, (2) setting daily movement goals and (3) posters with exercises and walking routes. Physical activity was measured with a movement sensor (PAM AM400) which measures active minutes per day.

**Main measures:**

Primary outcome was the mean difference in active minutes per day pre- and post-implementation. Secondary outcomes were length of stay, discharge destination, immobility-related complications, physical functioning, perceived difficulty to move, 30-day readmission, 30-day mortality and the adoption of the intervention.

**Results:**

A total of 61 patients was included pre-implementation, and a total of 56 patients was included post-implementation. Pre-implementation, patients were active 38 ± 21 minutes (mean ± SD) per day, and post-implementation 50 ± 31 minutes per day (Δ12, *P* = 0.031). Perceived difficulty to move decreased from 3.4 to 1.7 (0–10) (Δ1.7, *P* = 0.008). No significant differences were found in other secondary outcomes.

**Conclusions:**

The goal-directed movement intervention seems to increase physical activity levels during hospitalization. Therefore, this intervention might be useful for other hospitals to stimulate inpatient physical activity.

## Introduction

During hospitalization, patients are physically inactive.^1,2^ In general, they spend up to 23 hours per day sitting or lying in bed.^3^ This low amount of physical activity is associated with pulmonary complications and thrombosis.^4^ These are in turn associated with an increased length of stay and a higher risk of mortality even after hospital admission.^5^ Overall, in-hospital inactivity is associated with functional decline,^6^ defined as having difficulties in performing activities in daily life.

Despite several studies showing the adverse effects of inactivity during hospital stay, inactivity is still deeply rooted in the hospital culture.^7,8^ Therefore, multidimensional interventions have been developed to improve in-hospital physical activity. Studies evaluated the effectiveness of a multidimensional intervention to improve physical activity and found a reduced time spent in bed,^7–9^ less functional decline during hospitalization,^10–12^ shorter length of stay^7,11,12^ and more patients being discharged to home.^7^

Movement sensors are useful to objectively, continuously and remotely monitor patients in surgical populations.^13^ While they have mainly been used for monitoring for research purposes, they may also facilitate physical activity in a hospital ward environment.^14,15^ Movement sensors allow patients to monitor their own physical activity and provide them with feedback, which makes them effective in increasing physical activity.^16,17^ Therefore, an intervention to stimulate physical activity using a movement sensor was developed using Intervention Mapping^18^ (van Grootel. et al., 2023, under review). The intervention enables healthcare professionals and patients to have continuous access to the amount of physical activity per patient and to set personalized movement goals.

The primary objective of this study was to investigate the preliminary effectiveness of a goal-directed movement intervention, implemented in usual care on physical activity in hospitalized patients. The secondary objective was to evaluate the preliminary effectiveness of a goal-directed movement intervention, implemented in usual care on length of stay, discharge destination, the incidence of immobility-related complications, 30-day readmission, mortality, physical functioning at discharge and perceived difficulty to move at discharge. Furthermore, the adoption of the intervention from the perspective of patients and healthcare professionals was evaluated.

## Methods

The intervention was developed following the Intervention Mapping approach in collaboration with healthcare professionals^18^ (Grootel, van., et al., 2023, submitted). Next, the intervention was implemented as usual care at the pulmonology and nephrology/gastroenterology wards of the University Medical Centre of Utrecht, The Netherlands. The study protocol was assessed and approved by the local Medical Ethics Committee (study protocol number 22-537). Written informed consent was obtained from all included patients.

**Figure 1.**
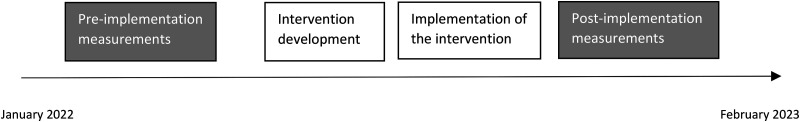
Timeline.

A pre–post design was used to evaluate the preliminary effectiveness of the intervention. Pre-implementation measurements were performed between April and June 2022. Usual care in the pre-implementation phase did not include movement sensors nor any other part of the intervention. Post-implementation measurements were performed between November 2022 and January 2023 (Figure 1). Patients who were admitted to the pulmonology or nephrology/gastro-enterology ward with an expected hospital length of stay of three days or more were eligible to be included in this study. Patients were excluded if they were wheelchair dependent, had a delirium, had a life expectancy less than one month or had language restrictions that made them unable to provide informed consent.

The Intervention Mapping approach was used to develop the goal-directed movement intervention. Intervention Mapping is an approach for the planning and development of health promotion interventions. In six steps, it maps the path from the recognition of a need or problem to the identification of a solution.^18^ The intervention included the following three main components: (1) Healthcare professionals had access to patients’ physical activity data via electronic patient records and a public screen on the ward. Patients had access to their own physical activity via a public screen on the ward and their personal mobile device. (2) Setting daily movement goals, with a standard baseline goal of 30 min. Healthcare professionals evaluated and increased movement goals by 5–10 min when a goal was achieved. (3) Posters with exercises and walking routes on the walls and floor of the wards. See supplementary materials for a detailed description of the goal-directed movement intervention. The implementation of the intervention also consisted of three main components (van Grootel. et al., 2023, under review): (1) Key users (experienced nurses) were assigned to build support under their colleagues, to embed the movement sensor in usual care and to answer questions about using the movement intervention, (2) Education sessions were organized and protocols for healthcare professionals were developed and (3) Physical activity data was incorporated into team meetings, for example, multidisciplinary consultations, progress and successes were shared at weekly meetings to encourage the use of the movement sensor and the amount of active minutes of the patients.

The senior nurse of the ward was consulted before approaching patients for participation in the study. Every patient who met the eligibility criteria was asked for participation in this study by a member of the research team (JvG and JN). If patients approved to participate in the pre-implementation measurement, they received a movement sensor. Post-implementation patients who received a movement sensor as part of usual care were asked to participate. Characteristics of the patients were retrospectively collected from the electronic patient record. The post-implementation measurements started when the following implementation goals were achieved: >70% of eligible patients wore the movement sensor and >50% of those patients had movement goals.

The primary outcome was physical activity measured in minutes per day using the Physical Activity Monitor (PAM) AM400. This ankle-worn movement sensor is a small button-shaped device which registers movements with an intensity of 1.4 METs and above. The active minutes are divided into light, medium and heavy intensity. The PAM has a strong agreement (ICC = 0.955) with the ActiGraph, a well-established activity monitor.^19^ Physical activity was measured continuously during hospital admission.

Secondary outcomes on patient level were length of stay, discharge destination (i.e., home or nursing home), the incidence of immobility-related complications (i.e., pneumonia, pulmonary embolism, deep venous thrombosis, urinary tract infection and pressure sores), 30-day readmission rate and mortality. Measurements of physical functioning were collected at admission (within two days after hospital admission, if possible) and at discharge (within two days before hospital discharge, if possible). Physical functioning was measured with the Activity Measure for Post-Acute Care ‘6-Clicks’ inpatient Basic Mobility at hospital discharge.^20^ This is a short form that has six items that were scored by the researchers on a 4-point ordinal scale and has an excellent reliability and validity in acute hospitalized patients.^21^ The total score ranges from 6 to 24 with higher scores indicating better function.^20^ In addition, the perceived difficulty to move was measured using a Numeric Rating Scale (NRS) ranging from 0 to 10.

Another secondary outcome was the adoption of the intervention of both patients and healthcare professionals using the Net Promoter Score for patients at hospital discharge. Healthcare professionals were asked to fill in the Net Promoter Score after the post-implementation measurements.^22^ The Net Promoter Score is based on a single question: How likely is it that you would recommend this intervention to a friend or colleague? Participants can give an answer ranging from 0 (‘not at all likely’) to 10 (‘extremely likely’).^22^ The assumption is that individuals scoring a 9 or a 10 will give positive word-of-mouth advertising; they are called ‘promoters’. Individuals answering 7 or 8 are considered indifferent and called ‘passives’. Finally, individuals answering 0–6 are likely to be dissatisfied customers and are labelled as ‘detractors’.^22^ The total score ranges from −100 to +100 and can be calculated by % promotors – % detractors, a score above 20 is considered ‘good/acceptable’, a score above 50 and indicates ‘great’ and a score above 70 indicates ‘excellent’.^23^ Experienced comfort while wearing the movement sensor is scored on an NRS from 0 to 10.

In addition, the following patient characteristics were collected: gender, age, body mass index, days of wearing the movement sensor, planned surgery (yes/no), restrictions (yes/no) (i.e., urinary catheter, thorax drain, intravenous infusion), pain and fatigue using an NRS from 0 to 10.

A previous study evaluating physical activity levels during hospital stay after oncological surgery, using the same movement sensor, found a mean of 37 active minutes per day, with a standard deviation of 13.^24^ An effect size of 0.21 was chosen based on previous studies.^24,25^ This resulted in a minimal sample size of 59 per measurement.^26^ Sample size analysis was conducted using Statulator: an online statistical calculator.^26^

Statistical analyses were conducted using IBM SPSS statistics software version 26 (IBM Corp). Data is presented and analyzed for the two wards as a whole. For a detailed presentation of the data per ward separately see supplementary materials. All continuous variables were tested for normality with the Kolmogorov–Smirnov test. Means were presented for normally distributed data and medians were presented for non-normally distributed data. Listwise deletion was used to handle missing data, all cases with missing scores on one or more variables are excluded from the analysis. Patient characteristics were described using descriptive statistics and tested with the Mann–Whitney *U* test or independent sample *t* test. For physical activity, minutes per intensity (i.e., light, medium or heavy) per day and the total active minutes per day were calculated. Differences in physical activity between pre- and post-implementation were tested using the independent sample *t* test. For secondary outcomes on patient level differences between pre- and post-implementation were also tested using the independent sample *t* test. Numbers and percentages were presented for the Net Promoter Score. The level of significance is set at *P* ≤ 0.05.

## Results

A total of 61 patients were included in the pre-implementation measurements and 56 patients were included in the post-implementation measurements. There were partially missing data for physical activity in 3 patients in the pre-implementation measurements and 5 patients in the post-implementation measurements. The main reason for missing data was non-wear (*n* = 2) and technical issues (*n* = 6). Table 1 presents the characteristics of the study population. See the supplementary materials for a detailed presentation of the data, stratified per ward. No significant differences were observed in the characteristics pre-implementation and post-implementation. Pre-implementation the mean wearing time was 6 ± 3 days, which was 46% of the total hospital admission days. Post-implementation, the mean wearing time was 7 ± 4 days, which was 58% of the total hospital admission days.

**Table 1.** Patient characteristics.

	Pre-implementation *n* = 61	Post-implementation *n* = 56	Independent sample T-test
Gender; *n* (%)			
- Male	31 (51)	31 (55)	.*627*
- Female	30 (49)	25 (45)	
Age; years, mean (SD)	60 ± 16	60 ± 14	.*908*
BMI; mean (SD)	24 ± 5	24 ± 5	.*854*
Planned surgery; *n* (%)	29 (48)	18 (32)	.*068*
Urinary catheter; *n* (%)	17 (28)	18 (32)	.*573*
Thorax drain; *n* (%)	19 (31)	16 (29)	.*812*
Intravenous infusion; *n* (%)	27 (44)	20 (36)	.*436*
Pain *admission* NPRS; mean (SD)	3 ± 3	3 ± 3	.*736*
Fatigue *admission* NRS; mean (SD)	6 ± 3	5 ± 3	.*054*
AM-PAC *admission;* mean (SD)	21 ± 4	22 ± 3	.*311*
Difficulty to move *admission* NRS; mean (SD)	5 ± 3	4 ± 3	.*502*

n: number; SD: standard deviation; BMI: body mass index; NPRS: numeric pain rating scale; NRS: Numeric Rating Scale; AM-PAC: acute measure for post-acute care.

### Physical activity

The mean of total active minutes per day increased from 38 ± 21 pre-implementation to 50 ± 31 post-implementation (Δ12, p = .031), an increase of 32%. The mean changes of physical activity per day per intensity level were light 29 ± 15 to 34 ± 15 (Δ5, *P* = 0.101), medium 9 ± 7 to 16 ± 21 (Δ7, *P* = 0.018) and heavy 0 ± 1 to 0 ± 1 (Δ0, *P* = 0.075) (Table 2). See Table 2 for detailed physical activity data.

**Table 2.** Physical activity outcomes.

	Pre-implementation *n* = 61	Post-implementation *n* = 56	*P*	95% CI Lower	Upper
Physical activity *minutes*; mean SD					
-Light	29 ± 15	34 ± 15	0.101	−10.355	0.933
-Medium	9 ± 7	16 ± 21	**0.** **018**	−13.624	−1.313
-Heavy	0 ± 1	0 ± 1	0.075	−0.584	0.028
-Total	38 ± 21	50 ± 31	**0.** **031**	−21.238	−1.057

SD: standard deviation; CI: confidence interval; *p*: *p*-value.

*P* < 0.05 indicates in bold.

### Secondary outcomes

Table 3 provides an overview of the secondary outcome measures on patient level. The difficulty to move at discharge changed from 3.4 points pre-implementation to 1.7 points post-implementation on a scale from 0–10 (Δ1.7, *P* = 0.008), exceeding the minimal clinically important difference.^27^ No significant differences were observed on other secondary outcome measures.

**Table 3.** Secondary outcomes.

	Pre-implementation *n* = 61	Post-implementation *n* = 56	*P*	95% CI Lower	Upper
LOS *days,* mean (SD)	13.0 ± 13.5	11.8 ± 12.6	0.638	−3.673	5.971
Discharge destination; *n* (%)					
-*Home*	59 (98)	51 (91)	0.201	0.144	0.031
*-Nursing home*	2 (2)	5 (9)			
Immobility-related complications; *n* (%)	18 (30)	10 (18)	0.140	−0.039	0.272
AM-PAC *discharge;* mean (SD)	22.9 ± 2.4	23.2 ± 1.3	0.417	−0.1021	0.426
Difficulty to move *discharge* NRS; mean (SD)	3.4 ± 3.2	1.7 ± 2.8	**0.** **008**	0.465	2.938
30-day readmission, *n* (%)	15 (25)	10 (18)	0.377	−0.083	0.218
30-day mortality, *n* (%)	0	0	–	–	

SD: standard deviation; LOS: length of stay; n: number; AM-PAC: activity measure for post-acute care; NRS: Numeric Rating Scale; CI: confidence interval; *p*: *p*-value.

*P* < 0.05 indicates in bold.

After the implementation, the adoption of the intervention was evaluated. Patients and healthcare professionals were asked how likely they would recommend the intervention to a friend or colleague. As a result of a protocol error, the Net Promoter Score of patients was collected retrospectively after discharge. In total, 13 (23%) patients responded. The mean score was 9 ± 1 and the total Net Promoter Score for patients was 46. Of all patients, 46% scored a 9 or higher and are considered ‘promotors’, 54% scored a 7 or 8 and are considered ‘passives’. Among healthcare professionals, *n* = 23 responded of which *n* = 19 nurses, *n* = 2 doctors and *n* = 2 physical therapists. Of all healthcare professionals, 31% scored a 9 or higher and are considered ‘promoters’, 65% scored a 7 or 8 and are considered ‘passives’, 4% scored a 6 or less and are considered ‘detractors’. The mean score was 8 ± 1 and the total Net Promoter Score for healthcare professionals was 26. The total scores of both patients and healthcare professionals are considered ‘good/acceptable’.^23^ Of all patients, n = 67 (57%) provided a score for the comfort of wearing the movement sensor. The mean score was 8 ± 2 (out of 10).

## Discussion

The purpose of this study was to evaluate the preliminary effectiveness of a goal-directed movement intervention on physical activity in hospitalized patients on two medical wards. This study adds to previous literature due to the novelty of an iterative process of intervention development, implementation and evaluation in usual care. To our knowledge, this is the first study evaluating the preliminary effectiveness of a movement intervention developed by an Intervention Mapping approach.

The results showed that post-implementation the mean level of physical activity was 12 minutes higher compared to pre-implementation (*P* = 0.031). This corresponds to an increase of 32% in physical activity per day. Perceived difficulty to move at discharge decreased from 3.4 to 1.7 points (Δ1.7, *P* = 0.008). There were no statistically significant changes in other secondary outcomes. This is in line with a systematic review that found behavior change techniques used in a movement intervention led to small to moderate increases in physical activity in hospitalized patients but did not show a significant effect on mobility or length of stay.^9^

Previous studies evaluating the effect of interventions using activity trackers mainly focused on the minimal clinically important change of step counts in patients with a chronic disease.^16,17^ Only few studies evaluated interventions with an activity tracker during hospital stay, whereby step count significantly increased.^28,29^ Another study evaluated a smartphone application with an activity tracker in hospitalized patients on standing and walking time.^30^ This study found an increase of 28 minutes (39%) in standing and walking, which was considered as clinically relevant.^30^ An increased time spend active seems important to reduce the risk of functional decline and postoperative complications.^10,16^ Therefore, the increase of 32% in active minutes per day found in this study seems relevant. However, guidelines on the recommended amount of physical activity do not yet exist^10,16^ and the question remains what amount of physical activity is needed to prevent functional decline.

Important active ingredients of the intervention are self-monitoring for patients, feedback of movement behavior and a multidisciplinary approach (van Grootel et al., 2023, under review). Therefore, the behavioral change techniques included in the intervention were feedback & monitoring, goals & planning and associations & antecedents. These behavioral change techniques proved to be successful in previous literature in non-hospitalized patients^9,17,31^ and are expected to have contributed to the change in physical activity. Additionally, research suggests that interventions using self-monitoring, among other behavioral change techniques, are more effective than those without.^32^ Furthermore, the integration of physical activity data in the electronic patient record is ensured because difficulties in integrating sensor data into the electronic patient record are a frequently reported barrier to implementation.^33^ Physical activity data is visible for healthcare professionals in usual care. Using activity trackers in usual care can support to achieve a common language regarding physical activity, which might enhance responsibility in the entire team.^34^

The perceived difficulty to move, scored on an NRS, changed from 3.4 to 1.7 (Δ1.7, *P* = 0.008) post-implementation. Although no literature is available on the minimal clinical difference in difficulty to move, literature stated that a change of 1.65 on the NRS for pain is a minimal clinically important difference in patients with acute pain.^27^ The change of 1.7 on the NRS might be clinically relevant, as it indicates that patients have less difficulty with performing activities such as transferring, walking and climbing the stairs. Furthermore, a previous study showed that higher physical fitness at discharge predicted better physical functioning at follow-up, in a surgical population.^35^ Besides this, the decrease in difficulty to move at discharge might lead to higher levels of physical activity which could contribute to a better recovery after discharge.^10^

Besides information about the effectiveness of the intervention, insight into the practical use of the intervention on the ward is needed for long-term change. Therefore, the adoption of the intervention by both patients and healthcare professionals was evaluated in this study. The Net Promoter Score for patients and healthcare professionals in this study is considered ‘good/acceptable’. This is in line with another study that investigated the usability of the software used in this study in an oncologic surgery population. This study stated the user experiences of patients were largely positive.^36^ Despite most of the users are satisfied with the intervention, the score might indicate that there is room for improvement to achieve long-term changes. To minimize the effort for healthcare professionals to use the movement sensor, the software platform used to link a movement sensor to a patient, to visualize physical activity data and to apply goal-setting was integrated in the electronic patient record. Nurses mentioned evaluating physical activity data with patients is mostly a task for physical therapists. Educating nurses in evaluating physical activity data and let them understand their role in this task might improve the adoption. Furthermore, during implementation, there were some technical issues when synchronizing patient data into the electronic patient record. Solving technical problems might improve the adoption.

An important strength of this study is the iterative process of intervention development based on previous literature and information from all stakeholders. Another strength is an evaluation in a real-world setting after a thorough implementation of usual care. There were also some limitations in this study. First, the measurements of the adoption and usage were restricted to the implementation period. To guarantee usage in the long term, structural evaluation is needed whereby the adoption and numbers of patients wearing the movement sensor should be measured. Second, due to a protocol error, there were many missing data in the evaluation of the adoption of the intervention from patients’ perspective. This could have led to an overestimation of the results. Third, a pragmatic pre–post design was used to evaluate the preliminary effectiveness. Therefore, the effectiveness of the intervention might have been influenced by other factors such as time and confounders. However, no significant differences were found in baseline characteristics. A pre–post design has also major advantages. Within the study design an iterative and dynamic process could be followed whereby the intervention was implemented in usual care. Hereby, the pre–post design provides an evaluation of actual change in usual care. The goal-directed movement intervention in hospitalized patients seems to be effective in increasing physical activity levels during hospital stay. Therefore, this intervention might be useful for other wards and hospitals to stimulate inpatients physical activity. More research is needed to investigate the effectiveness of the goal-directed movement intervention on outcomes after hospital discharge, such as recovery of patients in the long term.

**Clinical messages**

- The use of activity trackers in daily hospital care seems promising.
- A goal-directed movement intervention seems to contribute to an increase of physical activity in hospitalized patients.

## Supplemental Material

sj-docx-1-cre-10.1177_02692155231189607 - Supplemental material for Improving physical activity in hospitalized patients: The preliminary effectiveness of a goal-directed movement intervention Click here for additional data file. Supplemental material, sj-docx-1-cre-10.1177_02692155231189607 for Improving physical activity in hospitalized patients: The preliminary effectiveness of a goal-directed movement intervention by JWM van Grootel, P Bor and JA Netjes, C Veenhof, K Valkenet in Clinical Rehabilitation
