# Supplementary material for: Improving physical activity in hospitalized patients: The preliminary effectiveness of a goal-directed movement intervention
Source: Clin Rehabil. 2023 Jul 24;37(11):1501–9. doi: 10.1177/02692155231189607 (PMC10492426; doi:10.1177/02692155231189607)
Supplement: sj-docx-1-cre-10.1177_02692155231189607 - Supplemental material for Improving physical activity in hospitalized patients: The preliminary effectiveness of a goal-directed movement intervention [file sj-docx-1-cre-10.1177_02692155231189607.docx]

Supplementary materials

- Description of the goal directed movement intervention
- Patient characteristics stratified per ward
- Physical activity stratified per ward
- Secondary outcomes stratified per ward

| **Table:** Description of the goal directed movement intervention | | |  |
| --- | --- | --- | --- |
| **Intervention component** | **Explanation** | **BCT** | **Example** |
| 1. Feedback of movement behavior | Insight into the patients’ movement behavior via bedside tablets, the EPR and public screen on the ward. | Feedback & monitoring | *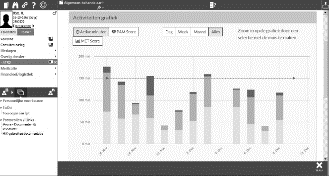* |
| 2. Goal setting | Healthcare professionals can set movement goals. A predefined movement goal is based on previous baseline measurements, 30 minutes on both wards in this study. | Goals & planning | 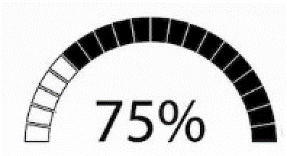  30 min |
| 3. Environment | Exercise posters on the walls. Walking routes on the floors (nudging). Educational posters in patients’ room. | Associations & antecedents | 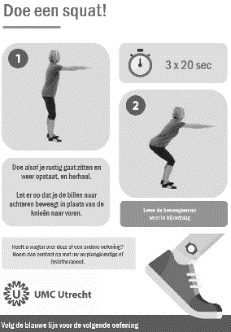 |
| Abbreviations: BCT: behavioral change technique, EPR: electronic patient record, HCP: healthcare professional | | | |
|  | | |  |

| **Table:** Patient characteristics | | | | | | | |
| --- | --- | --- | --- | --- | --- | --- | --- |
|  | **Pulmonology ward** | | | **Nephrology/Gastro-enterology ward** | | |  |
|  | Pre-impl.  n=28 | Post-impl.  n=26 | Mann-Whitney U | Pre-impl.  n=33 | Post-impl.  n=30 | Mann-Whitney U |  |
| **Gender**; n (%)   - Male - Female | 13 (46)  15 (54) | 14 (54)  12 (46) | *.589* | 18 (55)  15 (45) | 17 (57)  13 (43) | *.867* |  |
| **Age**; years mean (SD) | 61±15 | 65±12 | *.615* | 59±18 | 55±14 | *.250* |  |
| **BMI**; mean (SD) | 25±5 | 24±6 | *.340* | 23±5 | 24±5 | *.872* |  |
| **Planned surgery**; n (%) | 10 (36) | 2 (8) | ***.014*** | 19 (58) | 16 (53) | *.647* |  |
| **Urinary catheter**; n (%) | 3 (11) | 1 (4) | *.340* | 14 (42) | 17 (57) | *.207* |  |
| **Thorax drain**; n (%) | 7 (25) | 3 (12) | *.207* | 12 (37) | 13 (43) | *.501* |  |
| **Intravenous infusion**; n (%) | 13 (46) | 10 (39) | *.558* | 14 (42) | 10 (33) | *.596* |  |
| **Pain** *admission* NPRS; mean (SD) | 4±4 | 3±3 | *.907* | 3±3 | 4±3 | *.489* |  |
| **Fatigue** *admission* NRS; mean (SD) | 6±3 | 5±3 | *.111* | 5±3 | 4±3 | *.263* |  |
| **AM-PAC** *admission;* mean (SD) | 21±4 | 22±2 | *.167* | 21±3 | 21±3 | *.486* |  |
| **Difficulty to move** *admission* NRS; mean (SD) | 5±3 | 5±3 | *.257* | 4±3 | 4±3 | *.791* |  |
| Abbreviations: impl.=implementation, n=number, SD: standard deviation, BMI=body mass index, NPRS=numeric pain rating scale, NRS=numeric rating scale, AM-PAC=acute measure for post-acute care | | | | | | | |

| **Table:** Physical activity outcomes | | | | |
| --- | --- | --- | --- | --- |
|  | **Pulmonology ward** | | **Nephrology/gastro-enterology ward** | |
|  | Pre- impl.  n=28 | Post-impl.  n=26 | Pre- impl.  n=33 | Post-impl.  n=30 |
| **Physical activity** *minutes*; mean SD  -Light  -Medium  -Heavy  -Total | 28±17  8±6  0±0  37±22 | 36±17  17±25  0±1  53±37 | 30±13  10±9  0±1  40±20 | 32±12  16±17  0±1  47±26 |
| Abbreviations: impl.= implementation, SD=standard deviation, CI=confidence interval, *p*=p-value | | | | |

| **Table**: Secondary outcomes on patient level | | | | | | | | |
| --- | --- | --- | --- | --- | --- | --- | --- | --- |
|  | **Pulmonology ward** | | | **Nephrology/gastro-enterology ward** | | | |  |
|  | Pre- impl.  n=28 | | Post-impl.  n=26 | Pre- impl.  n=33 | | Post-impl.  n=30 | |  |
| **LOS** *days,* mean (SD) | 11.8±9.3 | | 8.4±8.6 | 13.9±16.4 | | 14.9±14.8 | |  |
| **Discharge destination;** n (%)  -*Home*  *-Nursing home* | 27 (96)  1 (4) | | 23 (89)  3 (11) | 32 (97)  1 (3) | | 28 (93)  2 (7) | |  |
| **Immobility related complications;** n (%) | 8 (30) | | 3 (12) | 10 (30) | | 7 (23) | |  |
| **AM-PAC** *discharge;* mean (SD) | 23.0±1.4 | | 23.1±1.3 | 22.8±3.1 | | 23.3±1.3 | |  |
| **Difficulty to move** *discharge* NRS; mean (SD) | 4.4±3.1 | | 2.7±3.1 | 2.4±2.9 | | 0.7±1,8 | |  |
| **30-day readmission** n (%) | 7 (25) | | 4 (15) | 8 (24) | | 6 (21) | |  |
| **30-day mortality** n (%) | 0 | 0 | | | 0 | |  |  |
| Abbreviations: impl.= implementation, SD: standard deviation, LOS:=length of stay, n=number, AM-PAC= activity measure for post-acute care, NRS= numeric rating scale, CI=confidence interval, *p.*=p-value | | | | | | | | |
